# Cancer Risk in HBV Patients With Statin and Metformin Use: A Population-Based Cohort Study

**DOI:** 10.1097/MD.0000000000000462

**Published:** 2015-02-13

**Authors:** Chang-I. Chen, Ching-Feng Kuan, Yu-Ann Fang, Shing-Hwa Liu, Ju-Chi Liu, Li-Li Wu, Chun-Jen Chang, Hsuan-Chia Yang, Jaulang Hwang, James S. Miser, Szu-Yuan Wu

**Affiliations:** From the Graduate Institute of Medical Science (C-IC); Center of Excellence for Cancer Research (C-IC, Y-AF); Cancer Center (C-IC), Wan Fang Hospital, Taipei Medical University, Taipei; Department of Health care Administration (C-FK), Central Taiwan University of Science and Technology, Taichung; Graduate Institute of Toxicology (S-HL), College of Medicine, National Taiwan University, Taipei; Division of Cardiovascular Medicine (J-CL), Department of Internal Medicine, Shuang Ho Hospital, Taipei Medical University, New Taipei City; Department of Ophthalmology (L-LW), National Taiwan University Hospital; Section of Endocrinology and Metabolism (C-JC), Department of Internal Medicine, Wan Fang Hospital, Taipei Medical University, Taipei; Institute of Biomedical Informatics (H-CY), National Yang Ming University; Department of Biochemistry (JH), School of Medicine, Taipei Medical University, Taipei, Taiwan; City of Hope National Medical Center (JSM), Duarte, CA; College of Medical Science and Technology (JSM), Taipei Medical University; Graduate Institute of Toxicology (SYW), College of Medicine, National Taiwan University, Taipei; Department of Internal Medicine (SYW), School of Medicine, College of Medicine; Department of Radiation Oncology (SYW), Wan Fang Hospital, Taipei Medical University, Taipei; Department of Biotechnology (SYW), Hung Kuang University, Taichung, Taiwan.

## Abstract

Chronic infection with hepatitis B virus (HBV) often causes chronic inflammation of the liver with an increased incidence of hepatocellular carcinoma (HCC). HBV-infected individuals may also have an increased incidence of nonliver cancers. Taking statin or metformin may decrease inflammation and infiltration, which may, as a result, reduce the risk of liver cancer or other major cancers in patients with HBV infection. The purpose of this study was to evaluate the hypothesis that statin and metformin could reduce the incidence of liver cancer (HCC) or nonliver cancers in patients with HBV.

Using the Taiwan Longitudinal Health Insurance Database 2000 to 2008, this cohort study comprised patients with a recorded diagnosis of HBV (*N* = 71,847) between January 1, 2000 and December 31, 2008. Each patient was followed-up until the end of 2008. The occurrence of HCC or a nonliver cancer was evaluated in patients who either were or were not taking statin or metformin. Cox proportional hazard regressions were used to evaluate the cancer incidence after adjusting for known confounding factors.

In total, 71,824 HBV-infected patients comprised the study cohort. Our study showed that either metformin or statin use was associated with a reduction in the incidence of cancer. This was most prominent in patients taking both statin and metformin. The adjusted hazard ratios (HRs) for patients using only statin were 0.52 (95% confidence interval [CI], 0.48–0.57) for all cancers, 0.28 (95% CI, 0.23–0.35) for liver cancer, and 0.63 (95% CI, 0.57–0.70) for nonliver cancers. Patients taking only metformin had risk-adjusted HRs of 0.82 (95% CI, 0.75–0.90) for all cancers, 0.97 (95% CI, 0.84–1.14) for liver cancer, and 0.75 (95% CI, 0.67–0.84) for nonliver cancers. A dose-dependent effect of statin use for chemoprevention was observed for all cancers, including both liver cancer and nonliver cancers. A dose-dependent effect of metformin was also seen in liver cancer and nonliver cancers without stratification into different cumulative daily doses of statin use.

This population-based cohort study investigated the protective effect of statin and metformin against cancer events in patients with HBV infection. Our study demonstrated that either statin or metformin served as independent chemopreventive agents with a dose–response effect in reducing the incidence of cancer with a dose–response effect of the agents and an additive or synergistic effect of combining statin and metformin use in reducing the incidence of many cancers.

## INTRODUCTION

Hepatitis B virus (HBV) infection is thought to play an important role in the pathophysiology of cancer. Possible reasons include a direct effect of HBV infection, changes in the host immune system as an effect of chronic infection, and behavioral factors associated with HBV infection. HBV results in not only hepatocellular carcinoma (HCC) but also nonliver cancers.^1^ Thus, chronically HBV-infected individuals may be at increased incidence of nonliver cancers. Medications that potentially reduce chronic inflammation, including statins and metformin, may reduce the risk of cancer in patients with chronic ongoing inflammation due to HBV.^2–10^ These alterations by statin or metformin use can affect the availability of structural lipids for the synthesis of membranes, the synthesis and degradation of lipids that contribute to energy homeostasis, and the abundance of lipids with signaling functions. Changes in lipid metabolism can affect numerous cancer cellular processes, including cancer cell growth, proliferation, differentiation, and motility.^11^ Lipid metabolism in cancers like HCC, colorectal, breast, lung, pancreas, and prostate has been discussed in many articles.^12–21^ Some studies suggested that the incidence of HCC in patients with HBV can be reduced by administering a statin and metformin;^22–24^ however, the protective effect of a statin and metformin against developing HCC or nonliver cancers in patients with HBV was not clearly demonstrated by those studies.

The mechanism by which metformin use decreases the incidence of nonliver cancers in HBV infected patients is not well understood. Some potential mechanisms have been investigated. The mechanism by which statin use decreases liver and nonliver cancer risk in HBV-infected patients is also still not well understood. The synergistic effect of the combined use of metformin and statin in reducing the risk of cancer has only been briefly discussed in the literature and limited to some specific cancers (eg, prostate cancer).^25^ But based on our data in animal models, administration of metformin and statin might enhance the therapeutic effect of local tumor through apoptotic and antiangiogenesis pathways. These results also seemed as the synergistic effect of statin and metformin combined use in tumor control.^26^ The aim of this study is to clarify the potential protective benefit of these drugs on the incidence of liver cancers or nonliver cancers in Taiwanese patients with HBV. We conducted a population-based cohort study using reimbursement claims from Taiwan's National Health Insurance (NHI) Research Database (NHIRD).

## METHODS

The NHI program has existed since 1995 to provide comprehensive health insurance coverage for all of Taiwan's residents. Currently, 98% of the >23 million people in Taiwan are covered under the NHI. This study used data from the NHIRD. There were no statistically significant differences in age, sex, or health care costs between the sample group and all enrollees. Data in the NHIRD that could be used to identify patients or care providers, including medical institutions and physicians, are scrambled before being sent to the National Health Research Institutes for database construction, and are further scrambled before being released to each researcher. Theoretically, it is impossible to query the data alone to identify individuals at any level using this database. All researchers who wish to use the NHIRD and its data subsets are required to sign a written agreement declaring that they have no intention of attempting to obtain information that could potentially violate the privacy of patients or care providers.

**FIGURE 1:**
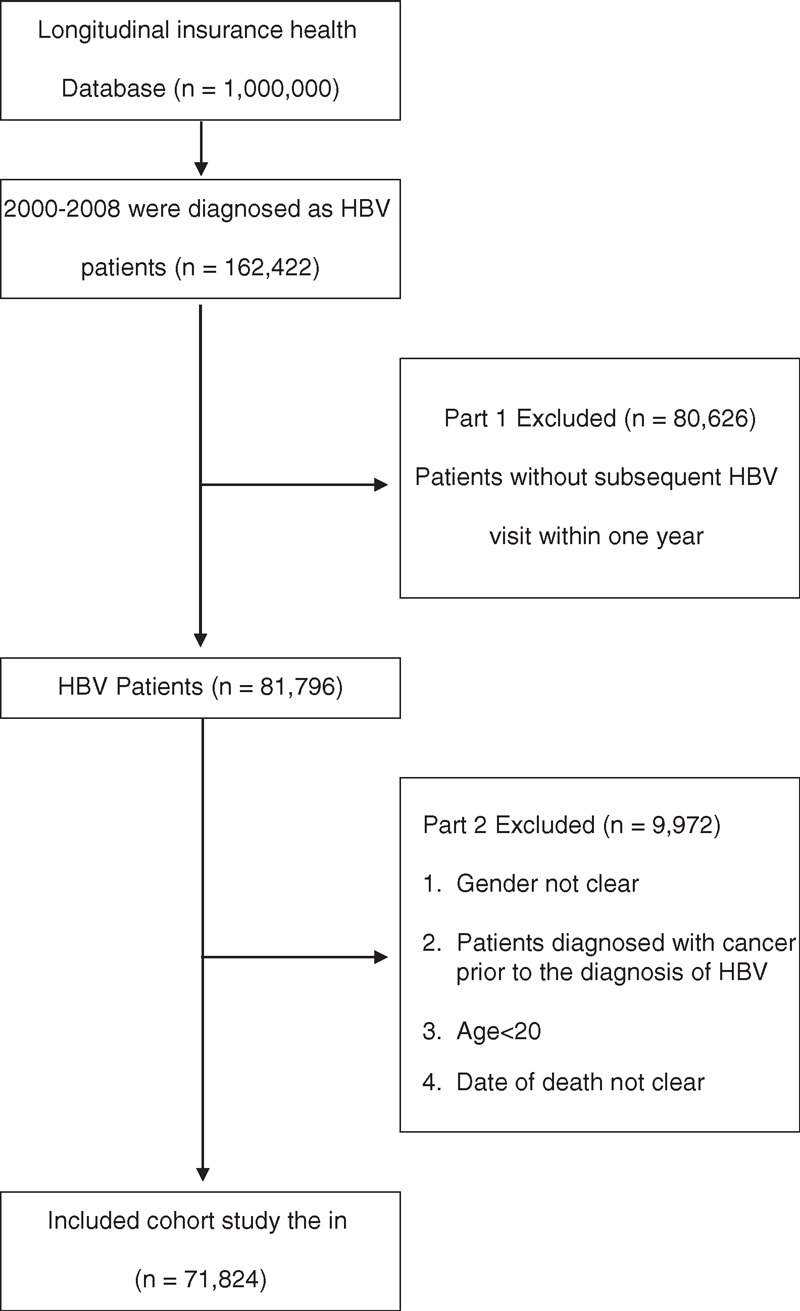
Flow chart of the selection of the cohort randomly sampled from a representative database from National Health Research Institute of Taiwan of 1,000,000 patients from the year 2005 registry of all NHI database for inclusion. HBV = hepatitis B virus.

The study cohort comprised all patients who visited health care facilities in Taiwan with a diagnosis of HBV (International Classification of Disease, 9th Revision, Clinical Modification Codes 070.2, 070.3, and V02.61) over a 9-year period (n = 162,422) from January 1, 2000 to December 31, 2008. All subjects without a subsequent outpatient visit or emergency visit for the diagnosis of HBV within 12 months were excluded (n = 80,626) because they were considered not to have chronic hepatitis disease (Figure 1). All subjects <20 years old on the day of diagnosis were excluded. We also excluded individuals who previously had been diagnosed with cancer prior to the diagnosis of HBV (n = 9972). Our final study cohort consisted of 71,824 cases of HBV carriers between 2000 and 2008 in Taiwan; 8861 were taking a statin only, 4774 were taking metformin only, 5121 were taking both a statin and metformin combined; and 53,037 were nonusers of either drug during the 9-year follow-up period. Each patient was tracked for the Charlson Comorbidity Index (CCI), the HCC risk, and all-cancer risk.

We identified patients who filled prescriptions for statins or metformin in the inpatient and ambulatory care order files between January 1, 1997, and 365 days before the index date for HCC. We collected dates of the prescriptions, the daily dose, the number of days supplied, and the number of pills per prescription.

The defined daily dose recommended by the World Health Organization is a unit for measuring a prescribed amount of drug. It is the assumed average maintenance dose per day of a drug consumed for its main indication in adults.^27^ To examine the dose–effect relationship, we categorized statins and metformin into 4 groups in each cohort (<28, 28–90, 91–365, and >365 cumulative defined daily dose [cDDD]) because the duration of the refill card was 3 months. Patients who used statins for <28 cDDDs were defined as statin nonusers.

### Statistical Analysis

Propensity scores are used as a statistical matching technique that attempts to estimate the effect of an intervention (statin/metformin) by accounting for the covariates (previously mentioned) that predict receiving the intervention (statin/metformin) and decrease selection bias. A polytomous logistic regression adjusted for the diagnosis age, sex, comorbidity condition, nonstatin lipid-lowering drugs, aspirin, acetylcholinesterase (ACE) inhibitors, area, index year, and anti-HBV drug was used. Because statins and metformin showed positive chemopreventive results, to examine potential effect modifiers, we conducted analyses stratified by groups with and without the use of statin or metformin. These sensitivity analyses were applied to evaluate the difference and consistency between the statins or metformin use and the risk of cancers.

## RESULTS

In total, 71,824 HBV-infected patients were included in the study cohort. Table 1 lists the demographic characteristics, medical conditions, and statin or metformin use by patients. Men were more commonly infected with HBV than women. Medication use by patients was related to age. A lower CCI was seen only in statin users. The distribution of CCI was more homogenous in metformin-only users. There were fewer HBV-infected individuals from the eastern region than from other regions of Taiwan.

**TABLE 1:**
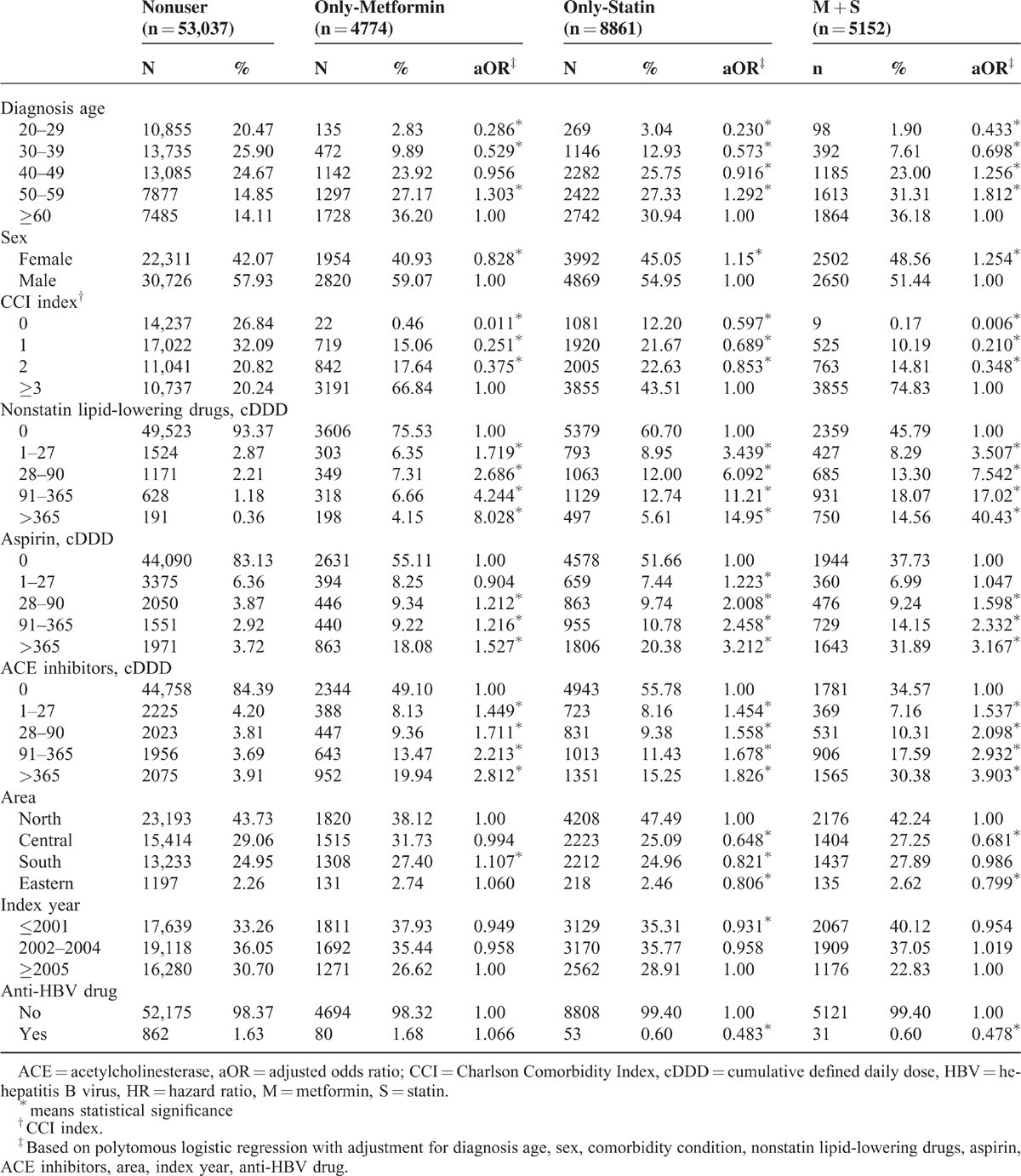
Characteristics of the Sample Population

Table 2 shows the incidence of all cancers associated with HBV, both liver and nonliver cancers, related to statin or metformin use. Our results show that statin use reduced the incidence of a variety of cancers. The most prominent reduction in cancer was noted in patients taking both a statin and metformin. Reduction of hazard ratios (HRs) in our study may suggest a synergistic effect of metformin added to a statin in all cancers except liver cancers. The use of both a statin and metformin in combination resulted in HRs that were smaller than those in the statin-only group.

**TABLE 2:**
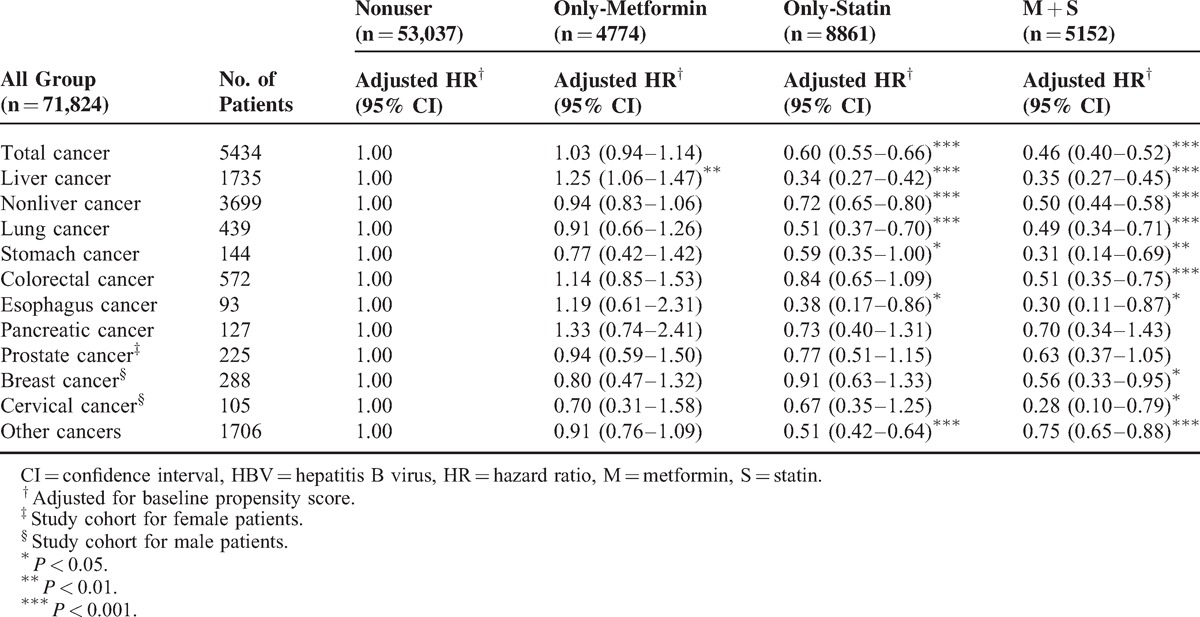
Risk of Overall and Individual Cancer With Statin or Metformin Use in HBV Patients

Data stratified by patient's factors are shown in Tables 3 and 4. The adjusted HRs for female HBV patients with metformin-use only were 0.78 (95% confidence interval [CI], 0.65–0.95) for nonliver cancers and 0.64 (95% CI, 0.47–0.88) for other cancers; however, the adjusted HR for liver cancer occurring in women with HBV using metformin did not show a significant reduction. Statin-only use by female HBV patients also reduced the incidence of all cancers and liver cancer. An increased protective effect was found in female patients with the combined use of a statin and metformin for colorectal, breast, and cervical cancers compared with statin-only or metformin-only use. This effect may be additive or synergistic. Lower HRs for all cancers, liver, nonliver, lung, and other cancers were also found in female patients with HBV infection.

**TABLE 3:**
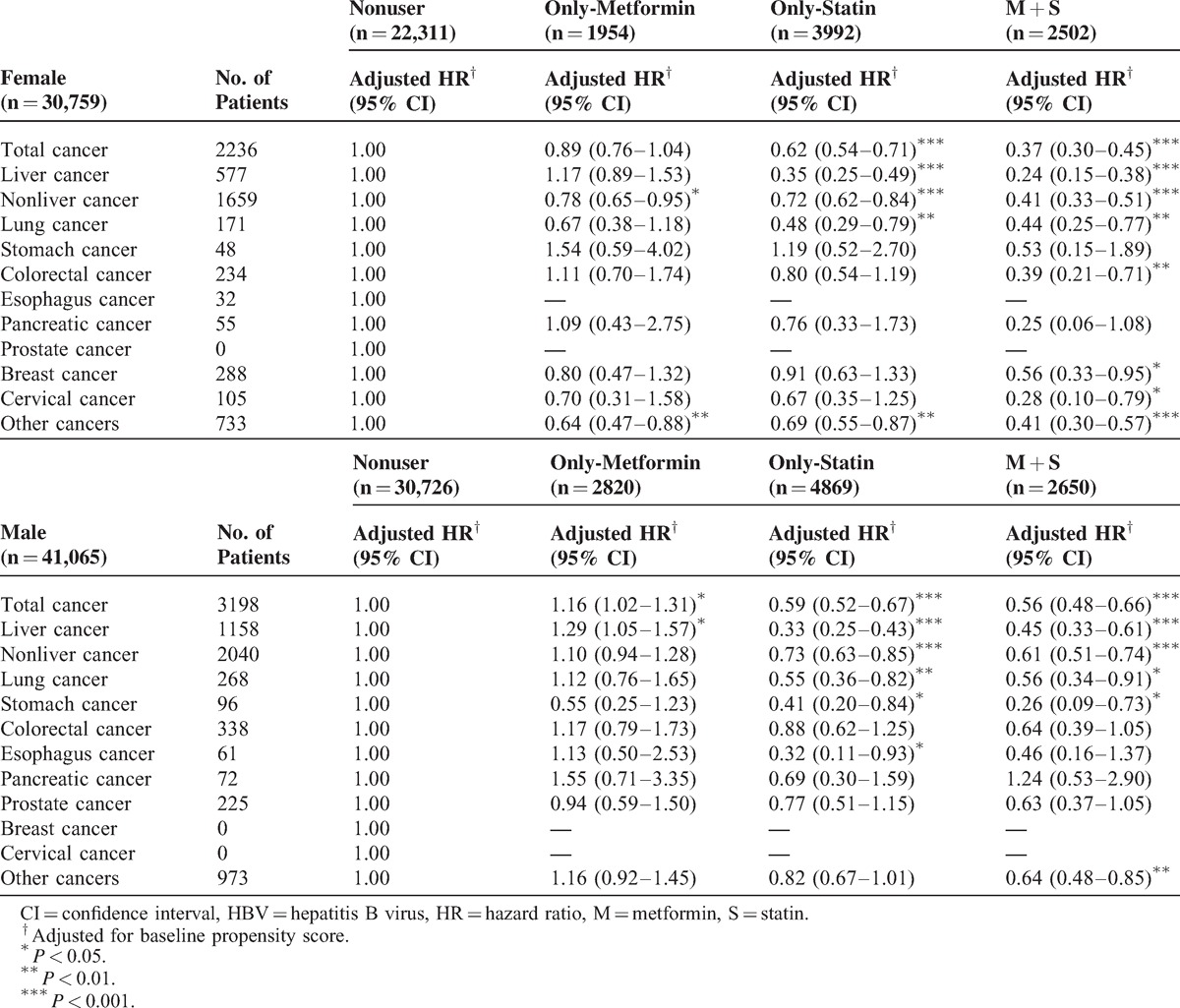
Risk of Overall and Individual Cancer With Statin or Metformin Use in HBV Patients Stratified by sex

**TABLE 4:**
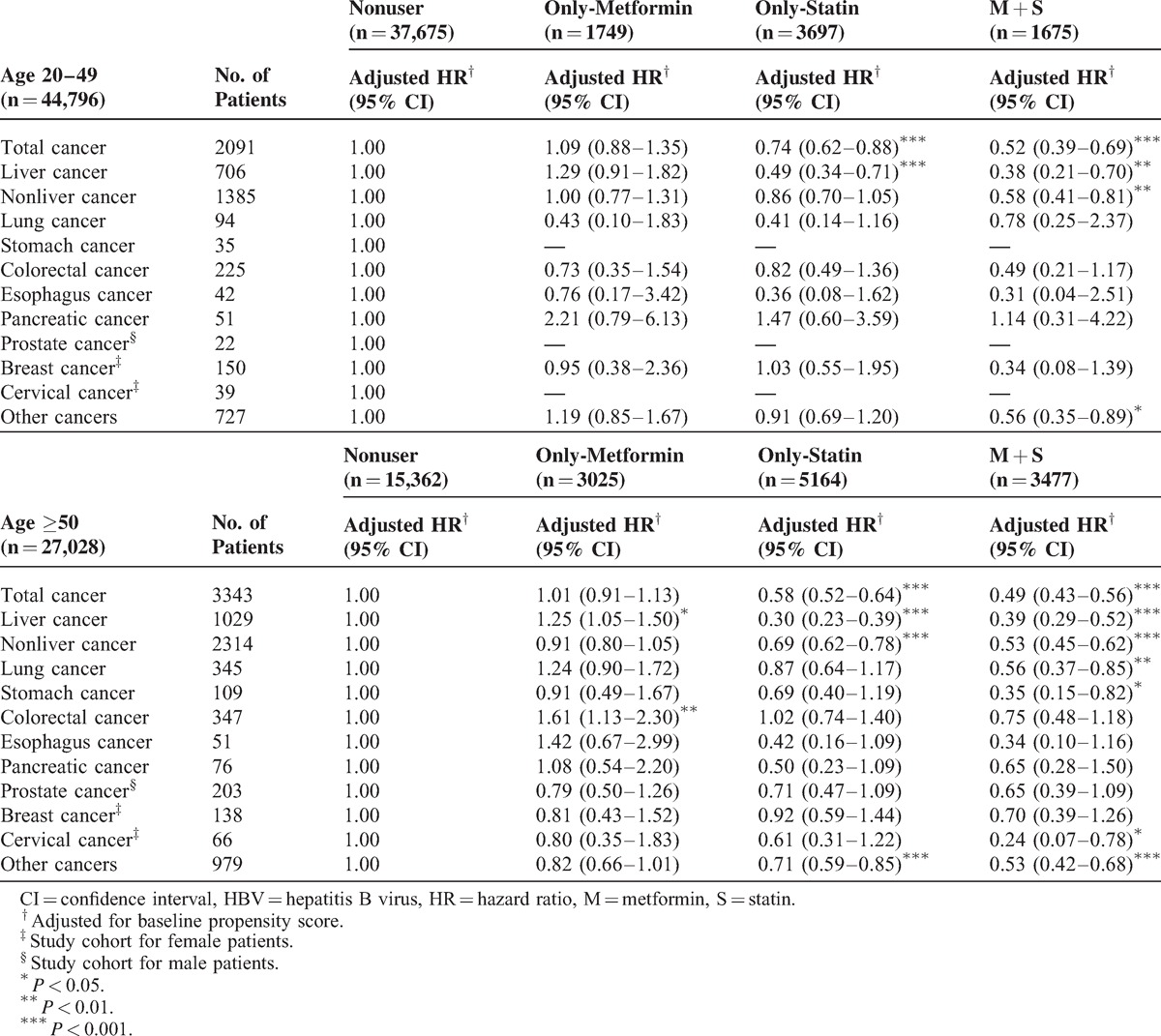
Risk of Overall and Individual Cancer With Statin or Metformin Use in HBV Patients Stratified by Age

For elderly patients with HBV infection, the combined use of metformin and statin resulted in lower HRs of total cancers, nonliver cancers, and other cancers. An additive or synergistic effect of the combined use of a statin and metformin was found for lung, stomach, and cervical cancers.

### Sensitivity Analysis

The sensitivity analysis adjustments had an effect on estimates of the association of statin and metformin use with the incidence of all cancers, liver cancer, and nonliver cancers in different models. Table 5 shows that the effects of statins remained significant in subgroups of different cDDDs of metformin use. When the data were stratified according to all cancers, liver cancer, and nonliver cancers analyzed, we still found a trend in the subgroup analysis. Persistent decreasing HRs directly related to increasing cDDDs of statin use were seen in the different cDDD metformin subgroups. *P* values for the trend within each subgroup were also significant. The dose-dependent chemopreventive effect of statin use existed in the all-cancer, liver cancer, and nonliver cancer groups. Table 6 shows the sensitivity analysis of adjusted HRs of metformin use in risk reduction for total cancers, liver cancer, and nonliver cancers during the follow-up period. The dose-dependent chemopreventive effect of metformin use existed in the total cancer group and in nonliver cancers without stratification into different cDDDs of statin use. The dose-dependent chemopreventive effect of metformin use existed for nonliver cancers with low to middle cDDDs of statin use. When metformin use was >365 cDDDs, the chemopreventive effect existed in the total cancer group and the nonliver cancer group with low to middle cDDDs of statin use.

**TABLE 5:**
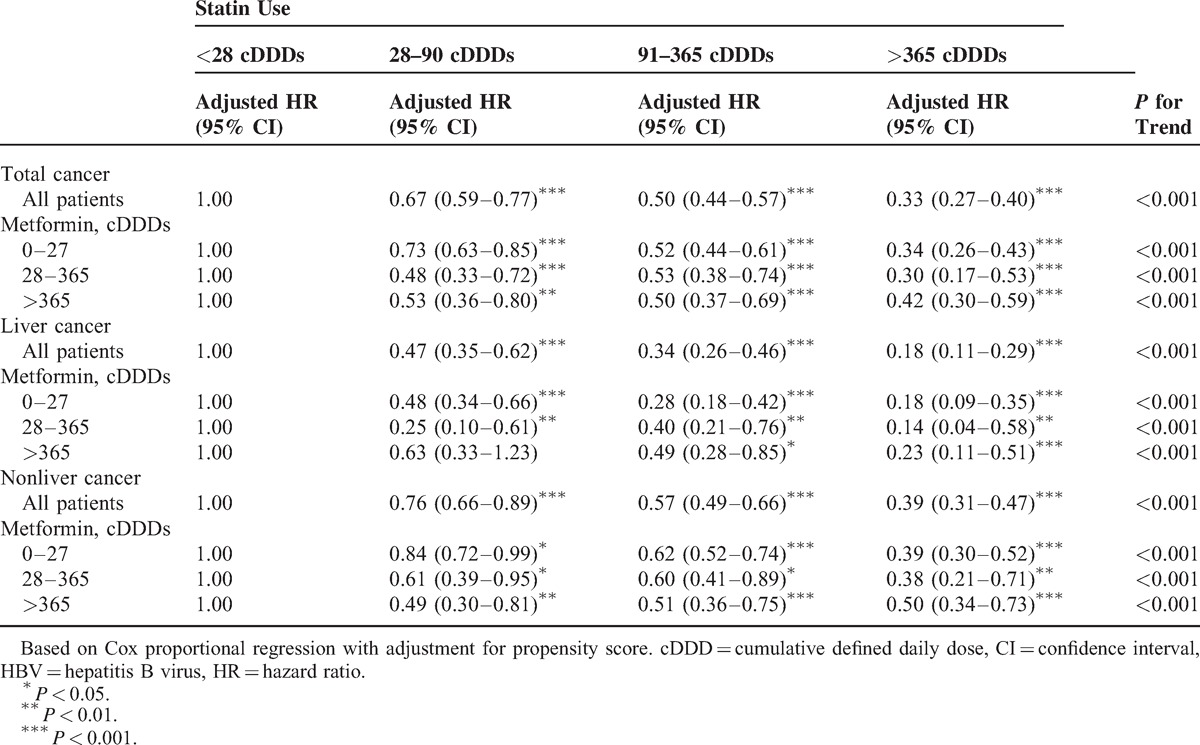
Sensitivity Analysis of Adjusted HRs of Statin Use in Risk Reduction of All Cancers, Liver Cancer, and Nonliver Cancers During the Follow-Up Period in the HBV-Infected Cohort

**TABLE 6:**
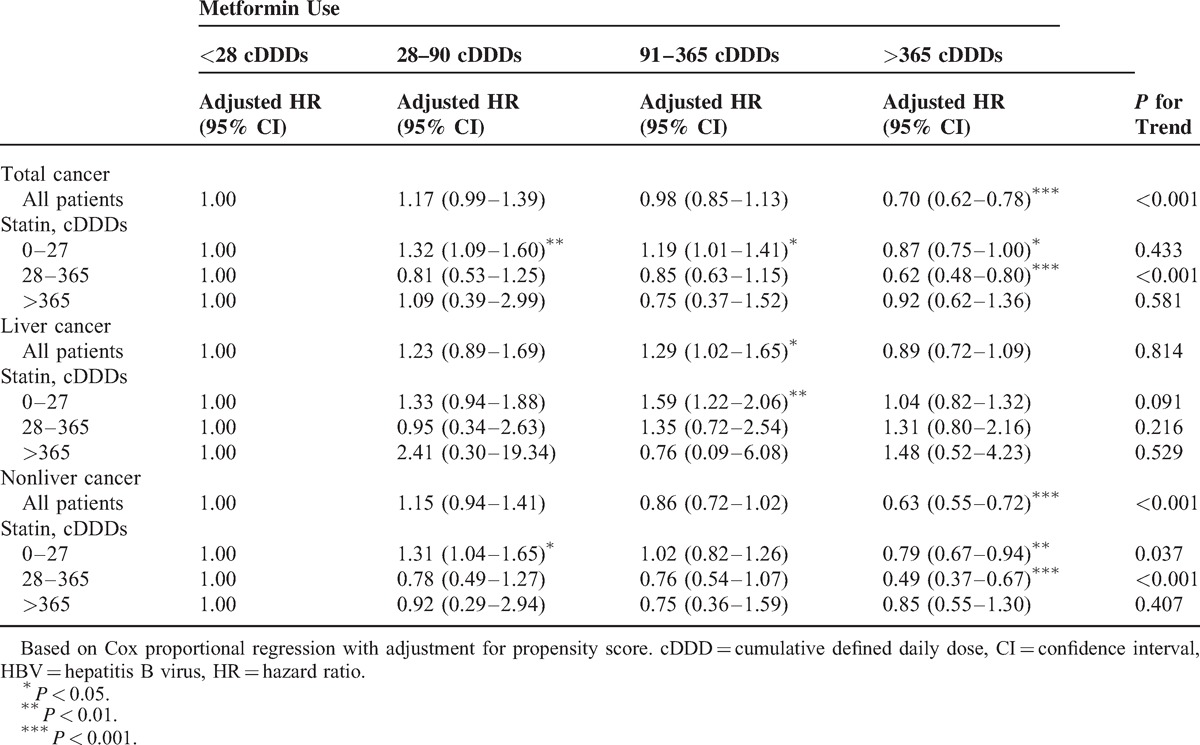
Sensitivity Analysis of Adjusted HRs of Metformin Use in Risk Reduction of All Cancers, Liver Cancer, and Nonliver Cancers During the Follow-Up Period in the HBV-Infected Cohort

## DISCUSSION

HBV is a very important medical and public health problem in Taiwan. HBV-related HCC was the second leading cause of death in Taiwan in 2008; however, HBV results in HCC and also nonliver cancers in endemic populations.^1^ Finding effective chemopreventive agents for this population is a major issue in Taiwan.

Many studies have suggested strategies to reduce the risk of cancer incidence.^22,28^ Data from a number of reports suggested that the incidence of HCC is reduced in type 2 diabetic patients who received metformin.^29–31^ The current study did not demonstrate a protective of metformin alone for liver cancer without stratifying for cDDDs, a result different from previous studies.^29–32^ Instead, our population differed from previous studies, and the dose-dependent effects of metformin use were not evaluated in their studies. It comprised patients with HBV infection. The study by Lai et al^32^ showed that after adjusting for sex, age, and comorbidities, patients with diabetes mellitus (DM), HBV, and HCV taking metformin had the lowest HCC HR at 0.49 (95% CI, 0.37–0.66), followed by patients taking thiazolidinedione (HR, 0.56; 95% CI, 0.37–0.84). Taking insulin, sulfonylurea, and α-glycosidase inhibitors also reduced the HCC risk; however, the reductions were not statistically significant. Prior studies showed that the high incidence of HCC in diabetic patients can be reduced by using metformin.^32^ In our study, metformin did not reduce the development of liver cancers (Table 6). Our data demonstrated that HBV carriers can be protected from developing liver cancer by statin use with a dose-dependent effect (Table 5). Further, metformin use can reduce the risk for nonliver cancers in HBV-infected patients. When stratified by cDDDs of metformin use, outcomes showed that high cDDDs of metformin use (>365 cDDDs) could significantly reduce the adjusted HR of nonliver cancers to 0.63 (95% CI, 0.55–0.72) (Table 6). Compared with previous studies, our data suggest that high cDDDs of metformin use can result in a significant protective effect against nonliver cancers. An additive or synergistic protective effect of the combined use of statin and metformin against liver cancer was not seen in our study and will require randomized clinical trials to investigate the hypothesis that there is a synergistic protective effect of the combined statin and metformin use against liver cancer.

Among postulated mechanisms for such a benefit are the inhibition of cancer cell growth and suppression of human epidermal growth factor receptor 2 overexpression and inhibition of mammalian target of rapamycin (mTOR).^33–35^ Metformin activates the AMP-activated protein kinase (AMPK) pathway, a major sensor of the energy status of cells. Metformin is also an inhibitor of mTOR catalytic activity, inducing a decrease in blood glucose by decreasing hepatic gluconeogenesis and stimulating glucose uptake in muscles.^36^ Several other potential mechanisms for suppressing cancer growth by metformin in vitro and in vivo include inhibition of protein synthesis,^37–40^ reduction in circulating insulin levels,^41–45^ inhibition of the unfolded protein response,^46,47^ activation of the immune system,^48,49^ and eradication of cancer stem cells.^50–54^ Our study also confirmed that the risk of total cancers and nonliver cancers in HBV infection patients taking metformin was decreased. The outcomes were comparable with those of other studies.^22,28^

The protective effects of a statin in our study of liver cancer were similar to the outcomes seen by Tsan et al.^55^ Statin use may reduce the risk of liver cancers in HBV-infected patients. The adjusted HR (0.34) (Table 2) in the current study was comparable with that in the study by Tsan et al^55^ (HR, 0.34). To our knowledge, this is the first article demonstrating that statin use can reduce the incidence of liver and nonliver cancers in HBV-infected patients.

Possible mechanisms for statin use decreasing the risk of cancer include inhibition of downstream products of the mevalonate pathway,^2,4–6^ triggering of tumor-specific apoptosis,^3^ inhibition of the proteasome pathway,^8^ and inhibition of cholesterol synthesis and HBV replication.^56^ Our study further showed that statin use decreases the risk of nonliver cancers including lung, stomach, colorectal, esophagus, prostate, and other uncommon cancers in HBV-infected patients.^25^

The synergistic effect of the combined use of metformin and statin in reducing the risk of cancer has only been briefly discussed in the literature and limited to some specific cancers (eg, prostate cancer).^25^ Given the possible synergistic effects of deregulated AMPK, RAS, and cholesterol biosynthesis pathways on cancer risk, the use of the combination may reduce cancer risk. Few studies have formally examined the interactive and potentially synergistic effects of the combination treatment with both drugs. This is the first article suggesting a synergistic protective effect of using both statin and metformin in patients with HBV infection. Because glucose metabolism is interrelated with lipid synthesis, the synergistic effect of metformin and statins on reduced cancer risk may be partly mediated by their joint lipid-lowering effect. The synergistic effect of the combined use of statin and metformin can be seen in colorectal cancer, breast cancer, cervical cancer in patients with HBV infection (Tables 2 and 3), nonliver cancers and other cancers in young (aged 20–49 years) patients (Table 4), and lung, stomach, and cervical cancers in older patients (age ≥50 years). Because statins and metformin may affect different pathways as chemopreventive agents, a synergistic effect may be seen; however, to prove this hypothesis, randomized studies with metabolic translational data are needed.

Our results showed that combined use of metformin and a statin had the greatest chemopreventive effect. Smaller HRs for all kinds of cancers were found. To test the potential dose–response relationship, we summed up the doses of statins and stratified statin use into <28, 28–90, 91–365, and >365 cDDDs (Table 5).^55^ The dose–response relationship of statin use existed for different cDDDs of metformin use, and there were significant *P* values for the cDDD trend of statin use. In recent years, increasing evidence has suggested a strong association between DM and HCC.^12,57–61^ Our data suggested that middle to high cDDDs of statin use is necessary to reduce the risk of liver cancer.

We also analyzed the potential dose–response relationship of metformin use. When metformin use was >365 cDDDs, the chemopreventive effect was the strongest. Although high cDDDs of metformin use may mean poor control of DM and may result in a higher incidence of cancers,^62–65^ high cDDDs of metformin use (>365 cDDDs) may still result in significant reductions in the risk of all cancers and nonliver cancers in patients with HBV infection. The data also suggest that statin and metformin were independent chemopreventive agents with dose–response effects in cancer prevention.

The strength of the present study is its large sample size. The results of our study suggest that the incidence of cancer in patients with HBV infection can be reduced by utilizing preventive strategies. This is also the first article that suggests a dose–response effect and synergistic effect of statin and metformin use in reducing the incidence of all kinds of cancers.

Potential limitations of this study should be noted. First, in recent years, DM appears to be a significant risk factor for developing several malignancies, including cancers of the breast, endometrium, pancreas, and liver.^63^ This may obscure the true value of these drugs. Second, several unmeasured confounders, including body mass index, smoking, alcohol intake, and other over-the-counter drug use, which are associated with cancers, were not included in our database. Third, we were unable to contact patients directly about their use of statins or metformin because the database did not include identifying data. Thus, we presumed that all prescribed medications were actually taken by patients as prescribed, which may have overestimated the actual ingested dosage, as some degree of noncompliance is always expected. Finally, because data on drug prescriptions were not complete in 1996, we could only evaluate statin and metformin use after 1997 because the use of these drugs before 1997 could not be captured for our analysis. This could have underestimated the cDDD and dose–response effects.

## CONCLUSIONS

This study is a population-based cohort study investigating the protective effect of statin and metformin against cancer events in patients with HBV infection. Our study further demonstrated that a statin and metformin were independent chemopreventive agents with dose–response effects in reducing the incidence of cancer. In addition to the dose–response effect, there appeared to be a synergistic chemopreventive effect of statin and metformin use for a number of different cancers. A prospective randomized trial evaluating the chemopreventive effect of a statin alone, metformin alone, and the combination is being developed.
